# How men receive and utilise partner support when trying to change their diet and physical activity within a men’s weight management programme

**DOI:** 10.1186/s12889-020-8213-z

**Published:** 2020-02-07

**Authors:** Sheela Tripathee, Helen Sweeting, Stephanie Chambers, Alice Maclean

**Affiliations:** 1 Institute of Applied Health Sciences, University of Aberdeen, Foresterhill, Aberdeen, AB25 2ZD UK; 2 MRC/CSO Social and Public Health Sciences Unit, Institute of Health and Wellbeing, University of Glasgow, 200 Renfield Street, Glasgow, G2 3AX UK

**Keywords:** Diet, Physical activity, Overweight/obesity, Weight, Couple, Football fans in training

## Abstract

**Background:**

The impacts of interventions designed to change health behaviours are potentially affected by the complex social systems in which they are embedded. This study uses Scottish data to explore how men receive and utilise partner support when attempting to change dietary practices and physical activity within the context of Football Fans in Training (FFIT), a gender-sensitised weight management and healthy living programme for men who are overweight/obese.

**Methods:**

Separate semi-structured face-to-face interviews were conducted with 20 men and their cohabiting female partners (total *n* = 40), 3–12 months after the men had completed FFIT. Data were thematically analysed and individual interviews were combined for dyadic analysis.

**Results:**

Men’s and women’s accounts suggested variations in men’s need for, and utilisation of, partner support in order to make changes to dietary practices and physical activity. There were also differences in descriptions of women’s involvement in men’s behaviour changes. Typologies were developed categorising men as ‘resolute’, ‘reliant’/‘receptive’ and ‘non-responsive’ and women as ‘very involved’, ‘partially involved’ and ‘not involved’. Men were more reliant, and women more involved, in changes to dietary practices compared to physical activity. The role of partner involvement in promoting men’s behaviour change seemed contingent on men’s resoluteness, or their reliance on the partner support.

**Conclusions:**

These results highlight how interactions between men’s resoluteness/reliance on cohabiting female partners and the partners’ involvement impact the extent to which female partners influence men’s changes to dietary practices and physical activity following a weight loss intervention. Understanding this interaction could increase the impact of health interventions aimed at one individual’s behaviour by considering other family members’ roles in facilitating those changes. The typologies developed for this study might contribute towards the development of behaviour change theories within the cohabiting couple context.

## Background

The worldwide prevalence of overweight and obesity tripled between 1975 and 2016 [1]. Of particular concern is the increase in overweight and obesity in men globally [2], as men are under-represented in weight-loss interventions [3]. Although the literature on men’s weight loss, and participation in weight loss interventions, has proliferated in recent years [4], evidence on the mechanisms behind men’s success (or not) in making weight related behavioural changes after participating in weight loss interventions is still limited.

A wide range of physical, psychological, sociological and environmental factors influence the adoption and maintenance of weight loss and associated health behaviours [5]. The literature in this area is diverse and brings together a number of theoretical perspectives [6]. Studies of weight-management, dietary changes and physical activity most frequently draw on psychological theories or models based on social cognition. Social cognition models are based on the social foundations of human learning, for example, models focusing on motivational factors underlying decisions to perform health behaviours, or on the processes by which goals are translated into action [7, 8]. Although these theories have been successful in predicting behavioural intentions, they have had limited success in predicting actual behaviour [9, 10]. In contrast to these psychological models, which focus on individual intentions, sociological perspectives conceptualise behaviours as individual and group performances of social practices [11]. Some of these social theories (e.g. Social Ecological models) [12, 13] position individual practices such as eating a healthy diet and being more physically active as fundamentally linked to their wider social context [14, 15]. Social support (provision or exchange of instrumental, emotional or informational assistance or resources that may arise from interpersonal relationships) is one aspect of social context that has been seen as helpful in making and maintaining behaviour change [6, 16, 17]. While this literature has extensively focussed on the *provision* of support [6, 16] and the types of support that can be provided [18–22], much less consideration has been given to how and if support is *utilised* by the ‘receiver’.

Family and cohabiting partners are important aspects of social context, influencing individual behaviours [23, 24] and impacting on behaviour change [6, 16, 25]. Consistent with this, studies focusing on men’s diet and/or physical activity have demonstrated that their health and health behaviours are inextricably tied to their family or household context [26, 27] and family members’ participation and support [28–31].

However, the evidence with regard to the influence of partner involvement specifically on men’s weight loss or health behaviour change is both limited and inconsistent [32]. Thus, while some studies indicate that involving family members, such as partners, as part of a weight loss intervention can positively influence men’s weight loss and weight loss maintenance [28], others have found men lose more weight when treated alone rather than with their partner [33] or that there are no differences in weight loss maintenance between those treated alone and with partners [3, 25]. Studies investigating the influence of female partners in men’s attempts to change dietary practices have found that men perceive the influence of their partner in their diet as significant [34–37]. With regard to partner influence on men’s physical activity changes, while some studies have shown that men are likely to be positively influenced by their female partner [38–40], other studies focusing on couple’s dyadic attempts to change physical activity have found that men are not influenced by their female partners [41] or that female partners can have a negative influence on men’s attempts to increase physical activity [42].

The existing literature (both theoretical and empirical) also underlines the importance of gender in relation to men’s weight and their attempts to change behaviours, and may help explain some of the inconsistent findings described above. Female prominence in food provision, associations between physical activity and masculinity [43], some masculine ideologies encouraging men’s unhealthy dietary habits, and stereotypical understandings of weight loss as feminine, are important gendered issues that can impact men’s weight loss and weight loss maintenance in the cohabiting couples’ context and have been highlighted in the literature [27, 44].

A number of previous studies have reported partners’ influence on men’s dietary changes following men’s diagnosis with an illness [35, 37, 45–47]. A men-only focus group study [36] explored the ways in which men’s efforts to change their eating practices were influenced by their female family members, including partners during and after a group-based, gender sensitised weight management programme (Football Fans in Training (FFIT). Consistent with prior research [34, 48, 49], this study found men described how their attempts to change their dietary practices required negotiations with female family members. Men reported that female family members responded in a range of ways to the changes men wanted to make, representing different levels of both positive and negative influences. However, performances of masculinity and femininity in relation to (healthy) men making dietary and physical activity changes within the cohabiting context, and the role they may play in men’s attempts to lose weight and maintain weight loss, have not been thoroughly explored from both partners’ perspectives.

The aim of the current study is to investigate how partner support is received and utilised by men trying to change their dietary practices and physical activity in order to lose weight through a weight management programme designed for obese/overweight men. Receipt and provision of support is explored from both partners’ perspectives, and the ways in which gender norms, roles and expectations are evident in participants’ accounts are considered, in order to facilitate deeper understanding of the complexities involved when behaviour change is attempted in the cohabiting couple context. This research provides new insights into the importance of the cohabiting context in the effectiveness of a weight loss intervention.

## Methods

### Recruitment

Forty participants (20 married or cohabiting [henceforth ‘cohabiting’] couples) were recruited through the FFIT programme at eight Scottish football clubs. The content and delivery of FFIT is described elsewhere [50]. In short, FFIT is a group-based ‘gender-sensitised’ weight management, physical activity and healthy living programme for overweight men aged 35–65. Men attend 12 weekly sessions at a professional football club, where they receive personalised advice and targets for changing their diet, participate in structured physical activity and are provided with tips on how to maintain the changes [51].

At the time of this study, FFIT was running at 32 Scottish clubs, from which all 13 club coaches who attended a FFIT annual meeting were approached to ask if they were able to support participant recruitment. Eight of these coaches agreed to provide this support. Of the eight clubs, three had already finished their FFIT sessions for that term. ST visited one FFIT session at each of the five clubs where the programme was ongoing and spoke to men about taking part in the study. All men present at these sessions completed the ‘permission to contact form’. In the three clubs where FFIT sessions were not ongoing at the time of recruitment, the coaches emailed an electronic ‘permission to contact form’ to men who had completed the programme.

Overall, 165 men completed and returned the ‘permission to contact form’. Of these, five said they did not want to be contacted, and 22 were ineligible because they were not cohabiting with a partner. All 138 eligible men who consented to be contacted were sent an information sheet about the study via email. They were asked to share the information with their cohabiting partner, and confirm if both of them were interested in participating. Recruitment stopped after 20 couples confirmed participation.

Discussion of qualitative sample sizes generally refers to ‘saturation’, when no new information or themes are observed. There is little advice on how to determine this in advance, but it will vary according to the research topic (focused/diffused) and aims, sample heterogeneity and data quality [52, 53]. The focused nature of the research questions and potentially rich data from interviews with each participant, together with the project timeline in mind, we aimed to recruit 20 couples to the study. This figure was flexible with the intention that more couples would be recruited if data saturation had not occurred after these interviews.

### Data collection

ST conducted separate semi-structured face-to-face interviews with each couple member between 3 and 12 months after the men had completed FFIT. Couple members were interviewed separately because exploring each partner’s perceptions or experiences in connection with the other (some potentially negative) was important for this study.

Most (*n* = 18) couples were interviewed one after each other; partners in two couples were interviewed on different days. Thirty-two interviews were conducted at participants’ homes, four in a university meeting room (with no one besides the participant and the researcher present) and four at local cafés (in spaces away from the public), according to participants’ preferences. Interviews were conducted between May and October 2016, and lasted on average 45 min (range 29–65 min).

Separate but linked/similar interview topic guides for men and women included questions on each partner’s: experiences of the man attending FFIT and of him making changes; men’s expectations and experience of receiving support from their partner, and their partners’ experience of providing support; reflections on maintenance of changes; and whether processes and experiences associated with making changes differed between dietary practices and physical activity. Participants were encouraged to talk about any important issues not covered by the topic guide. Full versions of the topic guides are available elsewhere [32].

### Data management and analysis

With participants’ written consent, interviews were audio recorded, transcribed verbatim, checked for accuracy and anonymised. NVivo version 11 was used to aid data storage and retrieval. A ‘Framework’ approach was used to manage the data and facilitate analysis [54].

All transcripts were read repeatedly by ST and a sample of transcripts was read by all authors. ST wrote a summary (paragraph) of each participant’s account and the participant’s profile for each couple based on the transcripts and the field notes. The memos were useful during analysis in aiding recall of participants and helping to clarify some of their remarks, thereby ensuring accurate interpretation of the data. Identified themes were discussed by authors in detail before coding data. Descriptive accounts were written based on the coded data and charted into framework matrices, which were reviewed by all authors. Each participant was assigned a row and each theme was presented in a column. Synthesising key categories and presenting them in matrices facilitated movement beyond descriptive accounts to provide explanations based on interpretations grounded in the data [55–57]. Additionally, both partners’ accounts were considered in each theme and coded for dyadic analysis [58]. This exercise facilitated exploration of each partner’s individual accounts, whilst considering the context of their shared life, to understand the basis of their experience and perceptions.

The framework approach was used to develop two typologies; one capturing men’s accounts of their levels of reliance on women’s involvement in order to make changes, and the other capturing women’s accounts of their levels of involvement in the changes men were making. While developing the typologies, men’s and women’s accounts of practices relating to diet and physical activity before, during and since men’s participation in FFIT were systematically analysed and compared (see Additional file 1). This process took into consideration who (man or woman) was reported as being practically involved in tasks and practices relevant to diet (e.g. meal planning, food preparation) and physical activity (e.g. arranging time to exercise), as well as how moral support around diet and physical activity was described.

## Results

### Sample description

All participants were white Scottish. They reported a range of occupations, indicating socio-economic variation across the sample. Participants’ ages ranged from 30 to 70 years (Mean 54 years); nine men were over age 60 (including two who were over the 65 year age limit set by FFIT when they joined the programme). The time that individual couples had cohabited ranged from four to 50 years. Seven men had attempted to lose weight prior to joining FFIT, only two of whom had followed formal weight-loss plans. Five men did not lose any weight while taking part in FFIT, but 11 lost 5% (generally regarded as clinically significant) [59] or more during the programme; by the time of the interview the percentage weight change since joining FFIT ranged from − 30% (lost 44 kg) to + 21% (gained 24 kg), with 14 reporting having lost 5% or more (Table 1). Seventeen women had previously attempted, or were at the time of the interview attempting, to lose weight.

**Table 1:** Sample characteristics

Men	Age range	Partners	Age range	Interview order	Cohabitation (years)	Time since FFIT (Months)	Men’s weight change compared to FFIT baseline			
							On completion of FFIT		3-12 months post FFIT	Total % lost from starting FFIT to interview
							Kg	%	Kg	%
Man #1	30-35	Partner #1^b^	30-35	F/M	8	5	0	0	+6	+3.9
Man #2	30-35	Partner #2^b^	30-35	M/F	4	11	-3	-3.1	-2	-5.2
Man #3	36-40	Partner #3^b^	30-35	F/M	18	5	-10	-9.0	-5	-13.5
Man #4	36-40	Partner #4^b^	41-45	F/M	8	5	0	0	0	0
Man #5^a^	41-45	Partner #5	41-45	F/M	21	12	-7	-7.1	+2	-5.1
Man #6	46-50	Partner #6^b^	51-55	M/F	10	12	-26	-17.8	-18	-30
Man #7^b^	51-55	Partner #7^b^	41-45	M/F	9	4	0	0	0	0
Man #8	56-60	Partner #8	51-55	F/M	30	4	-8	-7.5	+1	-6.6
Man #9^a^	56-60	Partner #9^a^	51-55	M/F	37	5	0	0	0	0
Man #10^b^	56-60	Partner #10^a^	51-55	F/M	5	4	-6	-4	-4	-6.7
Man #11^a^	56-60	Partner #11^b^	56-60	M/F	36	12	0	0	+24	+21
Man #12	61-65	Partner #12	56-60	F/M	34	6	-10	-9	+3	-6.3
Man #13	61-65	Partner #13^a^	61-65	M/F	44	12	-5	-6.2	-4	-11.2
Man #14	61-65	Partner #14	61-65	M/F	45	12	-14	-11.6	-4	-15
Man #15	61-65	Partner #15^b^	61-65	M/F	33	6	-22	-15	-16	-26
Man #16^a^	61-65	Partner #16^b^	61-65	F/M	44	3	-4	-4.6	-2	-7
Man #17	61-65	Partner #17^b^	66-70	M/F	50	12	-6	-6.7	-1	-7.8
Man #18^a^	66-70	Partner #18^b^	66-70	M/F	42	12	-16	-15.4	+3	-12.5
Man #19	66-70	Partner #19	61-65	M/F	40	7	-6	-7	-1	-8
Man #20	66-70	Partner #20	66-70	M/F	40	3	-3	-2.9	-2	-4.8

^a^had attempted to lose weight on their own prior to FFIT

^b^had participated in weight loss programme prior to FFIT

0 denotes no weight change; - denotes weight loss; + denotes weight gain

### Typologies of reliance and involvement

Analyses of both men’s and women’s accounts revealed three main findings regarding how partner support was utilised by men attempting to change their dietary practices and physical activity. Firstly, the men’s and women’s accounts suggested differences across the sample in terms of men’s need for, and utilisation of, partner support to make and maintain changes. Based on these accounts, men were categorised as: ‘*Resolute*’; ‘*Reliant*’ (diet)/‘*Receptive*’ (physical activity); and ‘*Non-Responsive*’ (Table 2). Secondly, the level of the women’s involvement varied across the sample, and women were therefore categorised as: ‘*Very Involved*’; ‘*Partially Involved*’; and ‘*Not Involved*’ (Table 3). Finally, except for Non-Responsive men who were Non-Responsive for changing both dietary practices and physical activity, levels of both men’s need for/utilisation of partner support and women’s involvement were not always the same for dietary practices and physical activity. Tables 4 and 5 show how men and women within each couple were categorised in relation to changes to dietary practices and physical activity respectively. Findings relating to if/how different ‘types’ of men’s attempts to make changes were influenced by the level of their partners’ involvement are discussed below for dietary practices and physical activity.

**Table 2:** Typology one: men’s responses to women’s involvement in their dietary practices and physical activity changes

Type of Man	Definition
Resolute	*N* = 9 (dietary practices); *N* = 6 (physical activity); *N* = 3 (both) • Made changes to practices without any support from partner. • Not dependent on partner but utilised her help to make dietary changes. • Preferred not to be coactive or have her practical involvement in physical activity changes.
Reliant	*N* = 7 (dietary practices); *N* = 0 (physical activity) • Dependent on partner for making changes. • Partner involvement was presented as key to these men making changes to diet.
Receptive	*N* = 0 (dietary practices); *N* = 10 (physical activity) • Receptive to partner involvement in their attempts to make changes, but not reliant on her support in order to do so.
Non-responsive	*N* = 4 (both) • Did not make changes, either independently or by utilising practical or moral support provided by partners. • Had intended to make changes on joining FFIT, but either did not do so or discontinued changes before the programme finished.

**Table 3:** Typology two: women’s level of involvement in men’s dietary practices and physical activity changes

Type of Woman	Definition
Very Involved	*N* = 8 (dietary practices); *N* = 6 (physical activity); *N* = 3 (both) • Provided extensive moral support and encouragement to help partner make changes. • Practically facilitated every aspect of partner’s changes. • Were codieting and/or coactive, and considered it their responsibility to help partner make changes.
Partially Involved	*N* = 9 (dietary practices); *N* = 10 (physical activity); *N* = 7 (both) • Involved in some aspects of partner’s changes, including providing both practical and/or moral support. • Did not consider it their responsibility to help partner make changes. • Some highlighted reasons for partial involvement (e.g. practical challenges or deciding to encourage partner to make changes independently in the hope that this would result in greater success/sustainability of changes).
Not Involved	*N* = 3 (dietary practices); *N* = 4 (physical activity); *N* = 2 (both) • Not involved in providing moral or practical support for any aspect of changes partner was making.

**Table 4:**
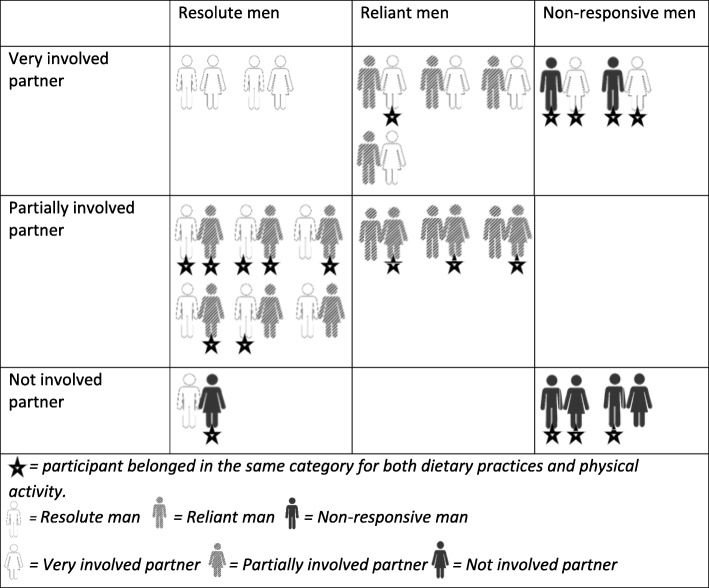
Couple reliance and involvement combinations for dietary changes

**Table 5:**
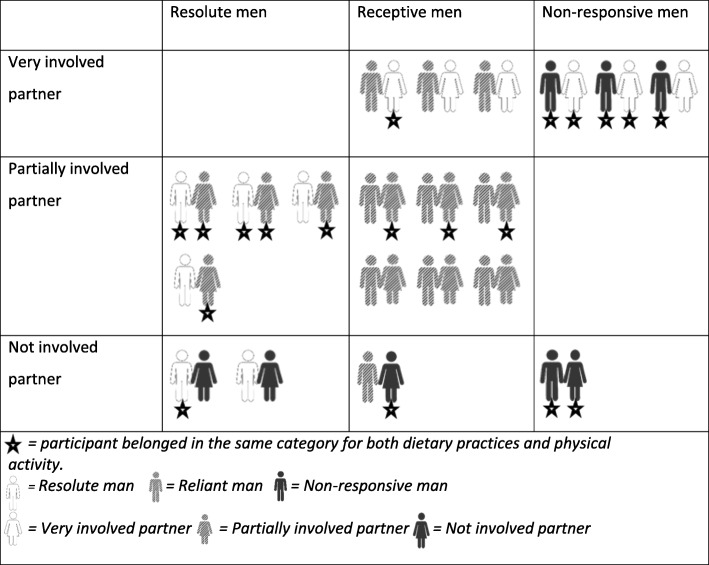
Couple reliance and involvement combinations for physical activity

### Making changes to men’s dietary practices: needs for, and utilisation of, partner support

#### Resolute men

Nine men were categorised as *Resolute* in respect of changes to their dietary practices. Their ability to make or maintain dietary changes was presented as not requiring partner involvement but any involvement from their partner was taken for granted and mostly described as facilitative to dietary changes. *Resolute* men and their partners reported that the man took charge of making and maintaining the changes himself and was determined to overcome any difficulties and discomforts while doing so. Most *Resolute* men presented themselves as either fully or partially involved in food-related activities in the household before joining FFIT. They described themselves as practically competent in making the necessary changes to their diet and suggested that they did not consider these to be their partner’s responsibility. Although *resolute* men spoke of accepting emotional and practical support from partners, such as if she offered to help cook, they framed help as something that their partner liked or chose to do rather than something they themselves needed.

*I think I had to […] try and do it myself [but] it definitely helped that she was eating healthier as well. (Man #12, age 61–65, cohabitation with partner #12 34 years.)*

*I was looking more at the food shopping with regards to my own healthy eating […] just really basically doing it on my own ‘cause she wasnae really involved that much. (Man #13, age 61–65, cohabitation with partner #13 44 years.)*

Although *Resolute* men’s involvement in family food practices suggests a lack of conformity to the traditional gendered division of food-related household labour, men’s and women’s accounts suggested that women’s involvement in dietary practices was still expected by these men and that a perceived lack of involvement required explanation. Where women were *Partially Involved* in *Resolute* men’s dietary changes, a justification was generally provided by both men and women, such as work schedules preventing the woman from providing more practical support, despite her wishing to do so, or the woman intentionally being less involved in order to facilitate the man’s autonomy. This suggests that the pressure of gender-related expectations may make women feel that they need to explain themselves for not fully conforming, even when their partner does not rely on their support.

*Because I was working full-time, he would do the cooking (Partner #12, age 56–60, cohabitation with Man #12 34 years.)*

*He didn’t expect me to get involved […] He is his own man […] If I could nag him or bully him in eating the way I wanted ...he’d not be my husband. He is who he is and so I can’t change him fully, but I like the way that he is changing himself and I support him in that. (Partner #8, age 51–55, cohabitation with Man #8 30 years.)*

In describing any lack of support from a *Partially/Not involved* partner in respect of changes to dietary practices, most *Resolute* men referred to ‘masculine’ traits in order to present themselves as independent, responsible and able to overcome problems. They commonly described themselves as ‘strong,’ when describing how they overcame challenges created by their partner’s lack of support.

*She is still buying crisps and chocolate when I was going through [dietary changes] which was a bit of a...I just had to be stronger myself, a bit disciplined. (Man #8, age 56–60, cohabitation with Partner #8 30 years.)*

### Reliant men

None of the seven *Reliant* men could be described as completely taking charge of any aspect of the dietary changes they were making or maintaining. While they did not necessarily make fewer dietary changes than *Resolute* men, they appeared less determined and motivated to make changes without partner support. This appeared less as an indication of lack of interest, and more as an expectation that their partner would or should be involved. Most of these men and women justified this expectation by providing either a practical rationale for the woman’s prominence, or suggesting barriers to greater involvement from the man. Examples included the woman’s habit of cooking for both, her being more skilled at cooking, or her work schedule making it more convenient for her to cook. Many *Reliant* men reported either having explicitly asked or expected their partner to help with making (specific aspects of) dietary changes. Almost all *Reliant* men expressed appreciation for their partners’ practical and moral support, and suggested this had determined the extent to which they had adopted and maintained healthy eating practices.

*She certainly drives what we eat […] I think if [partner name] hadn’t been doing that […] then I would have been a lot less successful. (Man #6, age 46–50, cohabitation with Partner #6 10 years.)*

Most *Reliant* men and their partners (comprising most of the couples aged over 60 years) suggested that the men were unable to make changes without partner support and some men also seemed to take their partner’s practical support for granted. Older partners of *Reliant* men explicitly described themselves as responsible for their partner’s dietary changes. In couples with a *Reliant* man and a *Very Involved* partner, both suggested the woman was in control of, and responsible for, the man’s diet. It was also evident that some couples were influenced by, and comfortable with, traditional norms around gender roles in relation to diet and food.

*My wife does it [food related work in the house] just she’s always done it. […] my mother always done the cooking [when growing up] I’d never do it [cook]. (Man #16, age 61–65, cohabitation with partner #16 44 years.)*

*Because when we got married, that was what you did. You were brought up to be like the home maker, and the men didn’t do that. (Partner #16, age 61–65, cohabitation with Man #16 44 years.)*

*Reliant* men and their *Partially Involved* partners suggested that both couple members were involved in making changes to the man’s dietary practices. However, although these men (mostly from younger couples) were involved in some aspects of dietary practices (e.g. menu planning, food preparation), what they ate was mostly driven by decisions made by their partner.

*He goes food shopping but I tell him what to buy. (Man #5, age 41–45, cohabitation with partner #5 21 years.)*

*Well, I’m eating the same as she makes. (Man #19, age 66–70, cohabitation with partner #19 40 years.)*

Partially Involved women talked about being supportive, and taking responsibility for meal preparation for their *Reliant* partner, mostly because they had no other choice as the man did not cook. However, unlike *Very Involved* women, they were generally not involved in encouraging their partner to eat healthy snacks, and were not codieting (purposefully changing their dietary practices together to eat healthily).

All *Reliant* men had depended on their cohabiting partner for dietary practices even before FFIT, so none had a *Not-Involved* partner for dietary practices.

### Non-responsive men

Four men were *Non-Responsive* for dietary practices, not making changes, regardless of level of partner support. Those with a *Very Involved* partner described having initially discussed as a couple the dietary changes that the man wanted to make, but he became disinterested in making changes soon after joining FFIT. These women described difficulty in convincing their partners to eat healthier options and explained how even their extensive involvement did not result in their partner changing his unhealthy practices due to his apparent lack of motivation. The *Very Involved* partners of *Non-Responsive* men suggested they had not given up on their attempts to encourage their partner to eat healthily.

*I will, make smoothies, I will make breakfast, I will do this for him, but then it’s his decision whether he has it or not and he doesn’t. (Partner #1, age 30–35, cohabitation with Man #1 18 years.)*

*Not Involved* partners of *Non Responsive* men described various reasons such as men’s lack of commitment to FFIT, and men’s indifference, for their own lack of support. In these cases, although women reflected on how their lack of support and modelling of unhealthy practices might have hindered his attempts and expressed some responsibility for his inability to make and maintain dietary changes, they also described why they did not, or could not, help their male partner.

*If he wants to do it [adopt healthy diet], I’ll (in future) encourage him to do it. But I don’t think you can force somebody to do something they don’t want to do. Because, they’ll end up disliking it even more and it’s not worth it. (Partner #11, age 56–60, cohabitation with Man #11 36 years.)*

### Making changes to men’s physical activity: needs for, and utilisation of, partner support

#### Resolute men

Accounts of the six men *Resolute* for changing physical activity suggested that they wanted to do this on their own, hence none had a *Very Involved* partner in relation to physical activity. These men often linked their lack of desire for *coactivity* (purposefully being physically active together) to their partner’s inability to do as much exercise as them. Aligned with dominant cultural ideals of masculinity that include physical prowess, self-reliance and independence, these men suggested how their partner joining them would ‘curtail’ the amount of exercise they wanted to do and described it as an inconvenience, unnecessary, or disadvantageous**.** Partners of these *Resolute* men also emphasised the importance of the man’s independent commitment to making changes to his physical activity, and constructed his ownership of the changes as essential for ensuring he made those changes.

*I don’t think it [coactivity] would have helped me more […] It wouldnae gave me any more encouragement because I was already totally into doing what I was doing. (Man #18, age 66–70, cohabitation with partner #18 42 years.)*

*I don’t think I needed to be any more supportive […] I just don’t push him to do it […] he’s got to do it for himself. (Partner #9, age 51–55, cohabitation with Man #9 37 years.)*

Although *Resolute* men and *Partially Involved* partners were *coactive* for some physical activity practices (such as walking), these men preferred their partner not to join them, and wanted to take charge of physical activity changes. They and their partners described the man exercising longer or doing more intense exercises than the woman.

All *Resolute* men and their partners also highlighted how the man’s desire to exercise alone could have resulted from both partners’ beliefs that the man was capable of making the changes without any support from the woman. The partners of *Resolute* men often expressed admiration for this resoluteness.

*I was quite impressed when he first started going [to FFIT] he was determined. (Partner #9, age 51–55, cohabitation with Man #9 37 years.)*

There were some similarities between how the *Partially* and *Not Involved* partners of men *Resolute* for physical activity changes, described their level of involvement (although *Partially Involved* partners were describing their lack of involvement in some aspects of the man’s changes and *Not Involved* partners were describing their lack of involvement in all of the changes the man was making). They often emphasised that each partner needed their own space. However, *Partially Involved* women did not appear to be completely unsupportive or indifferent, as they paid attention to whether the man was making and maintaining physical activity changes, and provided indirect support to accommodate these (e.g. freeing him from family obligations).

### Receptive men

While none of the men in this study were reliant on their partners for making changes to their physical activity, 10 men and their partners suggested the man was *Receptive* to their partner’s involvement. Most of these men described benefiting from coactivity, and the partner providing practical support and verbal encouragement to maintain his physical activities.

Most *Receptive* men with *Very Involved* partners for physical activity were coactive with partners. Some of these men described feeling obliged to include her, and framed themselves as a responsible partner in wanting to help her be healthier even though they “*would have to curtail (my) activity” (Man #14, age 61–65, cohabitation with Partner #14 45 years.).*

*Very* and *Partially Involved* women, including those who were not coactive, provided moral support to the man to help him change his physical activities. Examples included women verbally encouraging men to go out for exercise, praising their commitment to increasing their physical activities, and making it easier for them to take up additional activities. These couples described how the woman’s involvement (coactivity and moral support) encouraged and enabled the man’s attempts to increase certain physical activities.

*We used to enjoy (walking) as a stroll, but now there is more purpose beside it. […] And if he needs to make up his steps then we’d go out together later on until he reaches his steps count [target]. (Man #8, age 56–60, cohabitation with Partner #8 30 years.)*

*I walk further if she’s with me (Man #10, age 56–60, cohabitation with Partner #10 5 years.)*

A few *Partially Involved* partners of *Receptive* men attributed their lack of involvement to commitments such as work schedules, childcare arrangements, or their physical limitations. In these cases, both couple members described the circumstances as missed opportunities for *coactivity* rather than suggesting the woman’s absence was a favourable condition for the man’s changes. Some men and women also described women just showing an interest in what their partner was doing as encouragement in itself.

*I’d like to hope that I verbally encourage him [by] recognizing when he’s losing weight and kinda having those discussions. You know, listening when he came home from [playing] the football and talking about what he’s been doing. (Partner #3, age 30–35, cohabitation with Man #3 18 years.)*

*If she’d more time, she would go out and do more walking, but it’s just getting the time. (Man #10, age 56–60, cohabitation with Partner #10 5 years.)*

*I think it’s hard wae the kids, [otherwise] I’d love to do more exercise with [wife name]. (Man # 5, age 41–45, cohabitation with partner # 5 21 years.)*

Some *Partially Involved* women of *Receptive* men even framed their lack of direct involvement or verbal encouragement as a way to provide support, and not a sign of indifference toward the changes the man was making**.** Like the partners of *Resolute* men, these women often emphasised their respect for the man’s ability and desire to exercise independently, perhaps in line with feminine norms of care and nurture.

*It’s nice that he’s got more independence […] it’s not that we control each other, it’s just the way we’ve fallen in to the patterns over the years. (Partner #3, age 30–35, cohabitation with Man #3 18 years.)*

The only *Receptive* man with a *Not Involved* partner described her physical disability as the reason behind her lack of involvement. He said that although this did not affect his ability to make physical activity changes, he would have preferred to have her involvement or moral support. While his partner did not say why she refrained from providing moral support, she expressed guilt for being unable to be *coactive:* “[I felt] *bad for him that I cannae help him or cannae go wi’ him’ (Partner #15 61–65, cohabitation with Man #15 33 years.)*

### Non-Responsive men

The same four men who were *Non-Responsive* for changing dietary practices were also *Non-Responsive* for changing physical activity. Partners of Non-Responsive men expressed feelings of responsibility and wanting to help the men. Despite acknowledging the woman’s efforts, the men described lacking motivation to make any physical activity changes.

*She was trying to make me do that [be coactive], but I was stubborn […] I could have been doing the same thing as her, but I chose not to. (Man #1, age 30–35, cohabitation with Partner #1 8 years.)*

Accounts from couples featuring a *Non-Responsive* man and a *Very Involved* woman for physical activity suggested the man did not increase his physical activity even when she encouraged him to do so. These women expressed frustration about this.

*I do encourage him but he doesn’t really listen […] I think he thinks he doesn’t have time and that’s why, but he doesn’t realise that you have to make time. (Partner #1, age 30–35, cohabitation with Man #1 8 years.)*

The only *Non-Responsive* man with a *Not Involved* partner for physical activity described participating in physical activity at the FFIT sessions but not making any changes outside that setting. His partner, who described participating in some organised physical activities herself, reflected on her lack of involvement and suggested that being *coactive* could have encouraged him.

*Probably [my lack of encouragement] did hinder[...] whereas if I did [encourage him], he would have followed on a lot more […] maybe even saying, like, “Come on, let’s go out for a walk”. (Partner #4, age 41–45, cohabitation with Man #4 8 years.)*

## Discussion

This study has shown that female partners had different levels of involvement in men’s attempts to make changes to dietary practices and physical activity. Similarly, men exhibited different levels of reliance on partner support. These involvement and reliance dimensions facilitated an understanding of each couple’s unique context and the factors that bring about variations in outcomes for individuals [60] with regard to both dietary practices and physical activity. The positive influence a partner can have on men’s dietary changes following men’s diagnosis with an illness has been documented in previous studies, [35, 45–47, 49]. However, the evidence on whether healthy men benefit from partners’ involvement in changing dietary practices and physical activity is limited and disparate; this study adds to this evidence base.

Our findings relating to both men’s reliance on their partner for making dietary changes and variations in the level of partner involvement are consistent with prior research [34, 36, 48]. This article adds to this evidence, by showing that the influence which a partner’s level of involvement has on a man’s ability to make dietary changes is affected by his level of reliance on or responsiveness to her support. Consistent with previous studies following the man’s diagnosis of a chronic disease [49, 61], in the current study most couples’ food-related practices did not change remarkably as a result of men’s attempts to lose weight. Therefore, *Resolute* men who were already involved in family food provisions, such as cooking and shopping, were able to make changes to their diet autonomously. *Reliant* men continued to depend on their partner for dietary practices, meaning that their dietary changes were driven by their partner’s level of involvement. The partner’s level of involvement did not influence *Non-responsive* men’s dietary practices.

Our findings show that while partners of *Resolute* men were less involved, partners of *Receptive* men appeared to positively influence men’s attempts to increase their physical activity by facilitating their time to exercise alone, encouraging them, and/or being *coactive*. Limited evidence from previous quantitative studies suggests that a partner changing her physical activity as well as providing direct support, such as prompting the man to adhere to his physical activity goals, can be effective in helping men make changes [39, 40, 42, 62]. However, none of these studies provided explanations for *why* men did or did not make changes as a result of partner support. Our findings therefore lend further insights by showing that while partner involvement can facilitate physical activity changes among *Receptive* men, it may have less or no influence on those of *Resolute* or *Non-Responsive* men*.*

This study also found that the four men who were *Non-Responsive* were so for both dietary and physical activity changes, regardless of partners’ levels of involvement and use of various social control strategies [63]. Evidence on the positive or negative impact of support or control from a partner in making behavioural changes is inconsistent but it is reported that a partner’s support or control strategies generally *do* impact on an individual’s behaviour change outcome [64]. The current study suggests that a *Non-Responsive* person may not benefit from provision of support even within close relationship contexts.

The existing literature has reported on how men’s conformity to hegemonic masculinity could affect their health practices [35, 65, 66], and how female family members may impact their attempts to make changes to dietary practices [36, 67, 68]. Our findings suggest that gender role may contribute to *variation* in women’s influence on men’s changes to diet compared with physical activity. Both men’s need for support and partner involvement were greater for changing dietary practices, which are associated with femininity and women’s prominence, than in their physical activity, which is associated with masculinity. None of the men presented themselves as *Reliant* for making changes to physical activity and many men preferred not to be *coactive.* Therefore, unlike the female partners of men *Resolute* for dietary changes, who supported those changes by providing both moral and/or practical support, the partners of men *Resolute* for physical activity changes described being purposefully uninvolved, due to the man’s and/or their own lack of interest in their involvement. These descriptions also reflected men’s and women’s performances of gender as they emphasised masculine traits that helped men while also alluding to how the women helped by performing feminine roles, such as being caring and nurturing, as well as putting the man’s needs first and allowing them to be autonomous.

The literature in respect of Realist Evaluation has defined context as systems of interpersonal and social relationships [69] and argued that the fluidity of context, and its relationship with the mechanisms of behaviour change contribute to outcomes [60, 69]. The current study lends support to these theoretical perspectives in relation to cohabiting couples, by identifying how individual couple contexts influence the processes though which changes to men’s dietary practices and physical activity occurred within the same intervention (FFIT).

### Strengths and limitations

To our knowledge, this is the first qualitative study to explore the influence of ‘untreated’ female partners on healthy men’s dietary practices *and* physical activity following men’s participation in a weight loss programme. Most studies among healthy men have explored only men’s perspectives on partner influence [34, 36, 48]. Therefore, this study contributes to the literature on health practices in cohabiting couples’ contexts as well as on the complexities associated with each health practice. As the evidence regarding potential partner influence on individual practices is inconsistent, the comparison between the two practices in this study is important.

Recruiting all participants via FFIT provided the opportunity to explore the effectiveness of the same programme among different couple contexts. Additionally, it also allowed access to ‘healthy’ men who were at high risk of future diseases. Therefore, this article provides insights into a population and context that needs to be better understood to tackle obesity effectively, both on an individual and/or family level.

However, the findings also need to be considered against some limitations. All 20 male participants opted in to participate. This ‘self-selection’ means it is possible that FFIT participants who were less engaged with the programme were not represented. All 20 women in this study were recruited through their male partners, who therefore acted as ‘gatekeepers’ to both information about, and participation in, the study. Women who were unhappy with the partner’s participation in FFIT, or were disengaged from his behaviour change attempts may have been unlikely to participate. Additionally, the study did not consider whether participants’ health at the time of the study was a motivator for their efforts in making or supporting changes.

### Implications

This study has identified varying levels of both partner *involvement* in and men’s *reliance* on support. Further research is needed to understand if and how the influences described are maintained and/or impact over a longer time-period. As factors that reinforce maintenance of changed practices are likely to differ over time, a thorough understanding of how changed practices become habitual within the couple context is still needed. Given that a version of FFIT with minimal adaptations for women now exists [70], it could also be useful to compare the findings of this study with future studies among women FFIT participants, investigating the influence of male partners. This work would help to better understand the role of gender in partner influence on individual attempts to make behavioural changes.

The results of this study suggest several recommendations for future interventions, especially those wishing to engage partners in men’s weight loss interventions. Although men are more likely to attend and engage in programmes such as FFIT that are tailored for them and reinforce men’s ‘masculine capital’ [51], men in this study, still engaged with their partners and valued their support. Therefore, combining health interventions that are tailored for men with the provision of personalised advice on how best to solicit partner support, could provide an effective means of engaging men to adopt and maintain healthy practices. However, it would also be important to acknowledge variations in men’s reliance on (potential) partner support, since this may impact on the effectiveness of that support. Making efforts not to alienate female partners in designing weight loss interventions for men would be equally important. Prompting the *partners* to engage in supportive behaviours could be an effective approach in interventions targeting men.

## Conclusion

By demonstrating that men’s attempts to make changes to dietary practices and physical activity were influenced by both levels of partner involvement and men’s reliance on partners, the current study highlights the importance of the cohabiting context in the effectiveness of a weight loss intervention for healthy men. It suggests that combining health interventions tailored for men, which provide personalised advice on how best to solicit partner support, along with encouraging partners to find ways to engage in supportive behaviours, could be an effective approach in designing weight loss interventions for men. This is particularly important due to the long-term and interdependent nature of cohabiting couple relationships. These benefits could potentially apply to changes in practices other than diet and physical activity.

## Supplementary information

**Additional file 1.** Typology development. This table summarizes the comparison of participants to determine participants’ involvement/reliance categories for diet or physical activity.

## Data Availability

The qualitative data on which this article is based were collected by ST as part of her PhD research and are not publicly available. Further details of the study are available via http://theses.gla.ac.uk/id/eprint/41028 [32].

## Abbreviations

CSO: Chief Scientist Office
FFIT: Football Fans in Training
MRC: Medical Research Council
SPFL: Scottish Premier Football League
