# Supplementary material for: How men receive and utilise partner support when trying to change their diet and physical activity within a men’s weight management programme
Source: BMC Public Health. 2020 Feb 7;20:199. doi: 10.1186/s12889-020-8213-z (PMC7006401; doi:10.1186/s12889-020-8213-z)
Supplement: Supplementary file 1 — Additional file 1. Typology development. This table summarizes the comparison of participants to determine participants’ involvement/reliance categories for diet or physical activity. [file 12889_2020_8213_MOESM1_ESM.docx]

|  | **Responsibility for making the man’s dietary practice *healthier*** | | | | | | | | |  |
| --- | --- | --- | --- | --- | --- | --- | --- | --- | --- | --- |
| **Couples** | **Meal planning** | **Meals in the house** | **Food shopping** | **Food preparation** | **Outside meal** | **Healthy Snacks** | **In charge of changes** | **Partner’s moral support** | **Codieting** | **category** |
| **Man #14**  **Partner #14** | Both | Self | Both | Self | Both | Both | Self | Yes | Yes | **Resolute/**  **V Involved** |
| **Man #3**  **Partner #3** | Both | Both | Both | Both | Self | Self | Self | Yes | Yes | **Resolute/**  **V Involved** |
| **Man #10**  **Partner #10** | Self | Self | Self | Self | Both | Both | Self | Yes | Yes | **Resolute/**  **P Involved** |
| **Man #2 Partner #2** | Both | Self | Both | Self | self | Self | Self | Yes | Yes | **Resolute/**  **P Involved** |
| **Man #12**  **Partner #12** | Self | Self | Self | Self | Self | Self | Self | Yes | Partial | **Resolute/**  **P Involved** |
| **Man #13**  **Partner #13** | Self | Both | Self | Both | Self | Self | Self | Yes | No | **Resolute/**  **P Involved** |
| **Man #9* Partner #9** | Self | Self | Self | Both | self | Self | Self | Yes | Partial | **Resolute/**  **P Involved** |
| **Man #8**  **Partner #8** | Both | Her | Both | Her | Both | Self | Self | Yes | Partial | **Resolute/**  **P Involved** |
| **Man #15**  **Partner #15** | Self | Self | Self | Self | Self | Self | Self | No | No | **Resolute/**  **N Involved** |
| **Man #17 Partner #17** | Both | Her | her | Her | Both | Her | Her | Yes | Yes | **Reliant/**  **V Involved** |
| **Man #16**  **Partner #16** | Her | Her | Her | Her | Self | Her | Her | Yes | Yes | **Reliant/**  **V Involved** |
| **Man #6**  **Partner #6** | Her | Her | Her | Her | Self | Her | Her | Yes | Yes | **Reliant/**  **V Involved** |
| **Man #20**  **Partner #20** | Her | Her | Both | Her | Her | Her | Her | Yes | Partial | **Reliant/**  **V Involved** |
| **Man #5**  **Partner #5** | Both | Her | Both | Her | self | Both | Both | Yes | Partial | **Reliant/**  **P Involved** |
| **Man #19 Partner #19** | Both | Her | Both | Her | Self | Self | Both | Yes | No | **Reliant/**  **P Involved** |
| **Man #18 Partner #18** | Both | Her | Her | Her | Self | Self | Both | Yes | No | **Reliant/**  **P Involved** |
| **Man #1**  **Partner #1** | NR | NR | NR | NR | None | NR | Her | Yes | NR | **Non-responsive/**  **V Involved** |
| **Man #4**  **Partner #4** | None | None | None | None | None | None | None | No | No | **Non-responsive/**  **N Involved** |
| **Man #11* Partner #11** | None | None | None | None | None | None | None | No | No | **None-responsive/**  **N Involved** |
| **Man #7**  **Partner #7** | NR | NR | NR | NR | NA | NR | Her | Yes | NR | **Non-responsive/**  **V Involved** |

Additional file 1: Typology development: comparison of participants to determine participants’ involvement/reliance categories for diet or physical activity

|  | **Responsibility for increasing the man’s physical activity** | | | | | |  |
| --- | --- | --- | --- | --- | --- | --- | --- |
| **Couples** | **Walking** | **Gym** | **Leisure activity** | **Time arrangements** | **Partner’s moral support** | **Coactive** | **Category** |
| **Man #9* Partner #9** | Self | x | Self | x | Yes | Partial | **Resolute/**  **P Involved** |
| **Man #2**  **Partner #2** | Self | Self | self | Both | Yes | Partial | **Resolute/**  **P Involved** |
| **Man #19 Partner #19** | Both | Self | Self | Self | Yes | Partial | **Resolute/**  **P Involved** |
| **Man #16**  **Partner #16** | Self | Self | Self | x | Yes | No | **Resolute/**  **N Involved** |
| **Man #18 Partner #18** | Self | Self | self | x | Yes | No | **Resolute/**  **P Involved** |
| **Man #13**  **Partner #13** | Self | self | Self | Self | Yes | No | **Resolute/**  **N Involved** |
| **Man #6**  **Partner #6** | Both | Both | Both | x | Yes | Yes | **Receptive/**  **V Involved** |
| **Man #8**  **Partner #8** | Both | x | Both |  | Yes | Yes | **Receptive/**  **V Involved** |
| **Man #14**  **Partner #14** | Both | Both | Both | NA | Yes | Yes | **Receptive/**  **V Involved** |
| **Man #20**  **Partner #20** | Both | x | Self | Her | Yes | Partial | **Receptive/**  **P Involved** |
| **Man #5**  **Partner #5** | Self | Self | Self | Her | Yes | No | **Receptive/**  **P Involved** |
| **Man #12**  **Partner #12** | Self | x | Self | x | Yes | Partial | **Receptive/**  **P Involved** |
| **Man #10**  **Partner #10** | Both | Self | Both | Self | Yes | Partial | **Receptive/**  **P Involved** |
| **Man #3**  **Partner #3** | Self | Self | Both | Both | Yes | partial | **Receptive/**  **p Involved** |
| **Man #17 Partner #17** | Both | Self | Self | x | Yes | Partial | **Receptive/**  **P Involved** |
| **Man #15**  **Partner #15** | Self | Self | Self | Self | No | No | **Receptive/**  **N Involved** |
| **Man #1**  **Partner #1** | NR | NR | NR | NR | Yes | NR | **Non-responsive/**  **V Involved** |
| **Man #4**  **Partner #4** | None | None | None | None | No | No | **Non-responsive/**  **N Involved** |
| **Man #11* Partner #11** | Both | NR | NR | None | Yes | NR | **None responsive/ V Involved** |
| **Man #7**  **Partner #7** | NR | NR | NR | NR | Yes | NR | **Non-responsive/**  **V Involved** |
| * = Did not complete FFIT  V Involved = Very Involved, P Involved = Partially Involved, N Involved = Not Involved  NR = Non – responsive, X = Not relevant | | | | | | | |
